# Promising results in a 3-year follow-up for adults undergoing a one-stage surgery for residual talipes equinovarus as part of a humanitarian mission in Vietnam

**DOI:** 10.1186/s13018-022-03382-0

**Published:** 2022-11-16

**Authors:** Ezequiel Palmanovich, Wing Ip, Huynh Em, Jeffrey Spanko, Meir Nyska, Bruce Lehnert, Alex Tavdi, Nissim Ohana, David Segal

**Affiliations:** 1 Orthopedics Department, Meir Medical Center, Kfar Saba, Israel; 2 Sackler Faculty of Medicine, Tel Aviv University, Tel Aviv, Israel; 3 Community Medical Center, Stockton, CA USA; 4 Can Tho Central General Hospital, Can Tho City, Vietnam; 5 Veteran Affairs Palo Alto Health Care System, Palo Alto, CA USA; 6 S.O.A.R. Sport Orthopedics and Rehabilitation, Redwood City, CA USA

**Keywords:** Fixed rigid equinovarus, Low-technical surgical modalities, One-stage surgery, Humanitarian mission

## Abstract

**Background:**

Rigid talipes equinovarus (TEV) is a complex foot deformity in which the foot is fixed in a plantarflexed, inverted, and adducted position. This pathology has the potential to severely limit basic life activities, which can be devastating for patients in developing countries. The objective of this study was to present the outcomes of patients with mature bones presenting with severe rigid TEV deformity who were operated on during a humanitarian mission to Vietnam using a single lateral approach and a simple and inexpensive fixation technique.

**Methods:**

This is a retrospective analysis of prospectively collected data. We analyzed the outcomes of patients who underwent surgery for a severe rigid TEV that prevented them from walking minimal distances unaided. All feet were fixed in a non-plantigrade position. The surgeries were conducted as part of two International Extremity Project (IEP) missions in Can Tho, Vietnam (2013 and 2018). Pre- and post-operative AOFAS scores were compared using the paired sample t-test.

**Results:**

We operated on 14 feet of 12 patients, 6 (50%) of whom were males, aged 34.42 ± 11.7 (range 12 to 58). Four patients were followed for three months, two patients were followed for 12 months, and eight patients were followed for three years. On the final follow-up visit of each patient, all 14 operated feet were plantigrade with good alignment, and patients reported an improvement in daily activity. After 3 years of follow-up, the mean AOFAS score of eight patients with available data improved by 42.88 ± 3.91 points (95% CI 39.61 to 46.14, *P* < 0.01). Our patients also reported an improvement in mobility. At the final follow-up examination, no recurrence of the deformity was observed in any of the patients.

**Conclusions:**

Using low-technical surgical modalities, we were able to achieve plantigrade and walkable feet in patients with mature bones who had fixed rigid equinovarus.

**Level of evidence:**

Level IV- Case Series.

## Introduction

Talipes equinovarus (TEV) is a triple deformation (equinus, adduction, and supination) leading to an inversion of the foot. This deformity can be either flexible or rigid. Approximately 80% of children with congenital clubfoot are born in developing countries [1]. If left untreated, TEV can cause significant lifelong limitations. While the Ponseti method for treating congenital idiopathic clubfoot has been widely adopted [2], there are still areas in the developing world where this technique is not widely known or available. In many cases even if patients in these countries have been effectively treated during infancy, they are likely to develop rigid TEV because of the lack of follow-up. The most common causes of rigid TEV are neglected congenital clubfoot, poliomyelitis complications, post-traumatic residual deformity or chronic osteomyelitis [1]. Multiple scoring scales have been previously presented aiming to grade the severity of the deformity and to guide treatment [3]. Some have focused on clinical and/ or radiographic parameters [2, 4, 5], while others based their grading on clinical features, structural implications and reducibility [6, 7]. A common general principle is that while milder deformities (either graded by clinical features or reducibility) can lead to a wide range of gait disturbances, the more severe cases result in rigid non-plantigrade feet that are either un-shoe-able or un-walk-able [1, 8–11]. To achieve a plantigrade foot, these deformities require surgery [1, 8–11]. Furthermore, corrective surgery to restore foot alignment requires expensive hardware, such as internal or external fixation devices, as well as rigorous follow-up and rehabilitation regimens [1, 8–11] that are often inaccessible.

Following the introduction of the polio vaccine, poliomyelitis is now uncommon in the developed world. However, developing countries still bear the burden of this disease [12]. As a result, the population of these countries suffers most from poliomyelitis-related rigid TEV. The handicap caused by residual rigid TEV has the potential to be devastating to patients and their families. Due to long travel distances, limited commute options, and socioeconomic constraints, individuals often lack adequate access to proper medical facilities. Consequently, it is extremely difficult for patients in developing countries who have rigid TEV to undergo corrective surgery.

Some of the authors of this manuscript have spent the last few years participating in a humanitarian mission organized by the International Extremity Project (IEP) Group. TEV was a commonly encountered deformity in this project. The objective of this study is to present the outcomes of patients with severe rigid TEV deformity who were operated on using a single lateral approach and a simple and inexpensive fixation technique.

## Materials and methods

**Fig. 1:**
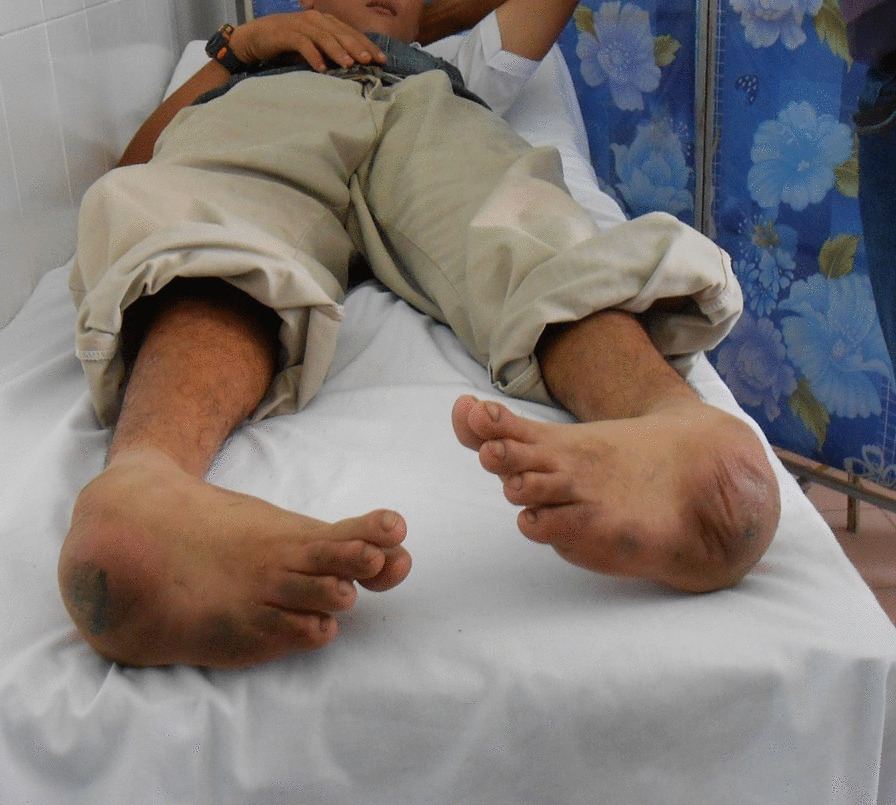
Neglected fixed non-plantigrade bilateral clubfoot in a 25-year-old patient

Following approval by the local hospital’s IRB Committee, we conducted a retrospective analysis of prospectively collected data. We analyzed data from patients with rigid TEV who underwent surgeries to correct severe non-plantigrade feet during two IEP missions to Can Tho, Vietnam (2013 and 2018). We included patients with severe fixed TEV deformity who were able to walk short distances despite their deformity. We excluded patients with cerebral palsy-related deformity and non-ambulatory patients (Fig. 1). Eventually we analyzed the data from 12 patients (14 feet), 6 of whom were male (50%). The mean age was 34.42 ± 11.7 (range 12–58) years. These patients have not been treated for their TEV deformity prior to the current surgery. Before the procedure, all patients were unable to walk a short distance outside without assistance, and their feet were fixed in a non-plantigrade position (Fig. 1). Patient demographic data are presented in Table 1.

**Table 1:** Demographics, diagnosis and operative plan of patients who underwent surgery for rigid talipes equinovarus (*n* = 12)

No	Age (years)	Gender	Pathology	Deformity	Side	Surgery
1	38	M	Post—polio deformity	Equinovarus deformity	Bl	Multiplanar osteotomy and TAL
2	31	F	Post—polio deformity	Equinovarus deformity—first ray elevatus	R	R 1^st^ ray dorsiflexion osteotomy + multiplanar osteotomy and TAL
3	33	F	Post—polio deformity	Equinovarus deformity	L	L lateral wedge STJ fusion with FHL and peroneal tendon transfer to calcaneus
4	12	M	Post—polio deformity	Equinovarus deformity	L	Multiplanar osteotomy and TAL
5	36	M	Post—polio deformity	Equinovarus deformity	L	Multiplanar osteotomy and TAL
6	39	F	Post—polio deformity	Equinovarus deformity	R	Multiplanar osteotomy and TAL + peroneal tendon stabilization
7	25	M	Neglected clubfoot	Equinovarus deformity	R	Multiplanar osteotomy and TAL
8	43	M	Neglected clubfoot	Equinovarus deformity	Bl	Multiplanar osteotomy and TAL
9	58	F	Neglected clubfoot	Equinovarus deformity	L	Multiplanar osteotomy and TAL
10	23	M	Neglected clubfoot	Equinovarus deformity	R	Multiplanar osteotomy
11	44	F	Neglected clubfoot	Equinovarus deformity	R	Multiplanar osteotomy and TAL
12	31	F	Neglected clubfoot	Equinovarus deformity	R	Multiplanar osteotomy and TAL

*Bl* bilateral, *F* female, *FHL* flexor hallucis longus, *L* left, *M* male, *R* right, *STJ* subtalar joint, *TAL* tendo-achilles lengthening procedure

The pre-operative evaluation was performed at the local hospital by a team comprised of IEP’s foot and ankle fellowship-trained surgeons, local orthopedic surgeons, and the missions’ physical therapist. The mission’s surgeons communicated with the patients in their native language, with assistance from local surgeons. A combination of thorough patient history, physical examination, and x-ray radiographs was used to identify the pathologies and possible treatment options. Upon admission, each patient received a score using the AOFAS scale. When surgery was considered the best treatment option, the surgical team provided the patients with an informed consent form detailing the nature of the planned procedure, post-operative cast, rehabilitation process, potential complications, and alternative treatment options. Informed consent was obtained in the patient’s native language.

**Fig. 2:**
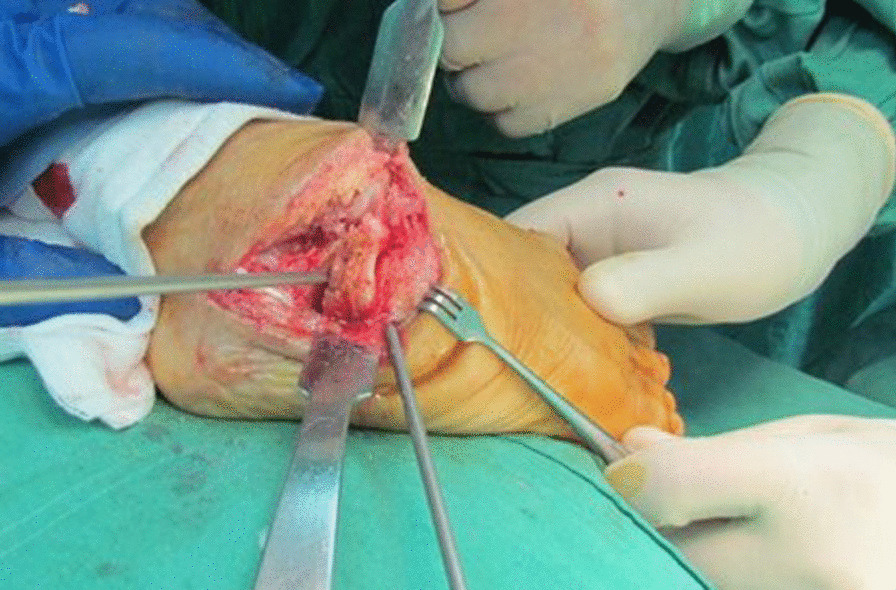
Fixed deformation during surgery. Note the wedge removal

**Fig. 3:**
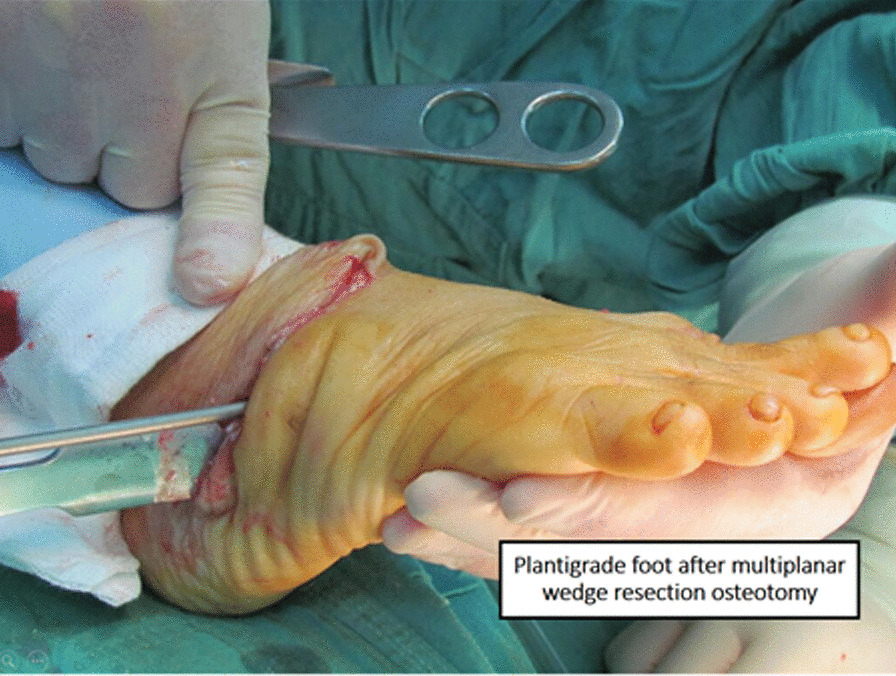
Plantigrade foot after wedge resection

The procedure was carried out under either general or spinal anesthesia. We utilized a dorsolateral approach comprised of a sinus tarsi approach extended plantarly to form a “J” cut. This was expanded to the subperiosteal level. To achieve a plantigrade foot, a multi-planar closing wedge osteotomy was performed based on the COR (center of rotation, i.e., center of the deformity) (Fig. 2). The osteotomy consisted of two non-parallel cuts, one proximal and one distal. The proximal cut was directed perpendicular to the non-deformed axis proximal to the COR, through the cuboid or anterior calcaneal process and directed further on toward the talus. The distal cut was made perpendicular to the non-deformed axis distal to the COR through the cuboid and the navicular. The wedge was removed, and the foot was realigned to its intended plantigrade configuration (Fig. 3). Since the wedge passed through the talonavicular and the calcaneocuboid joints, the final X ray resembled a triple arthrodesis. Reciprocal planning was performed as needed to achieve maximal contact between the bones. This osteotomy was utilized to correct the equinus component of the deformity, since the COR corresponded with the center of the equinus in the midfoot. In cases where the osteotomy was not sufficient to correct the equinus, a tendo-achilles lengthening (TAL) procedure was performed (10 out of 12 (83.3%) patients, Table 1). When both procedures were not sufficient, a posterior release could be added (none in the current patient population). Fixation was performed using three to four Steinmann wires. Fluoroscopy was unavailable in the operating theater. Soft tissue and skin were closed with absorbable sutures, and a below-knee circular cast was fitted. In two instances, gastrocnemius tendon resection was performed on the contralateral foot due to a mild similar deformity. These contralateral feet were not included in the analysis.

**Fig. 4:**
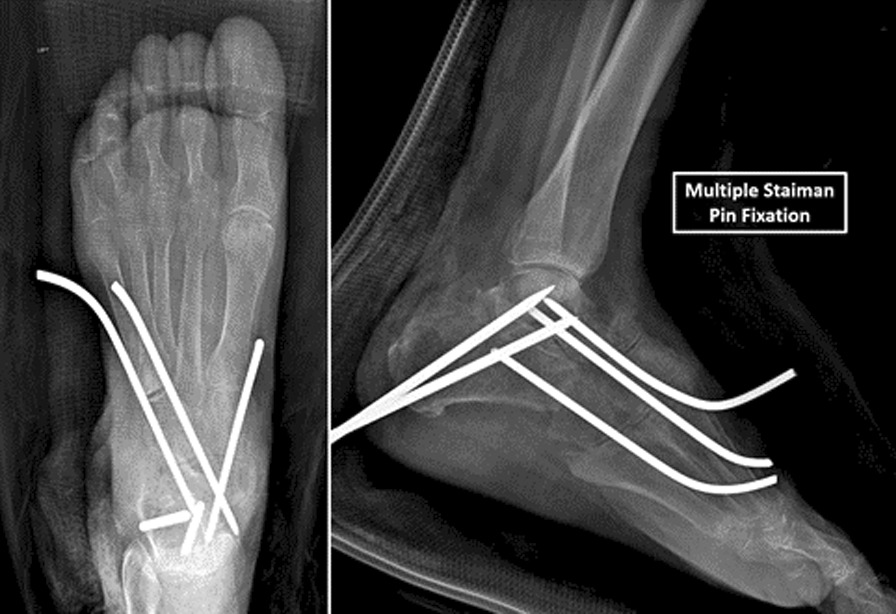
Postoperative x-ray image. Multiple Steinmann pin fixation is seen. In this case, subtalar joint fusion was also performed

**Fig. 5:**
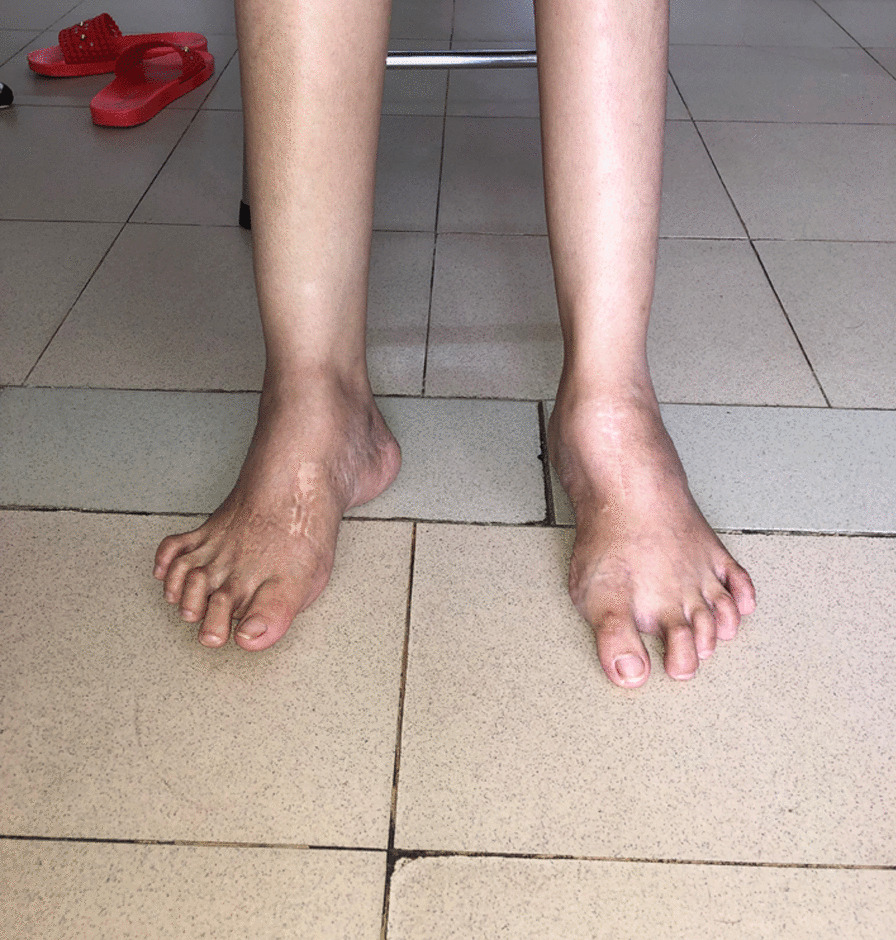
Clinical result at 3-year follow-up

Postoperatively, patients were hospitalized for a few days to a week to avoid long-distance travel immediately after the procedure and evaluate short-term complications. Due to limited access to medical care, the team was conservative with weight-bearing restrictions, prohibiting unprotected weight-bearing for three months. The first follow-up visit was scheduled for one week after the procedure, and an evaluation was conducted by a joint team of surgeons from the mission and the local hospital. During subsequent follow-up visits, evaluations were performed only by the local surgical team. The Steinmann pins were removed six to eight weeks following surgery, and full weight-bearing in a walking boot was permitted (Fig. 4). Since our patients often lived far from the hospital, continuous postoperative evaluations were limited. None of the patients were able to undergo personalized physical therapy, and no systematic radiographic follow-up was available. Successful treatment was defined as achieving a plantigrade foot that allowed unassisted and relatively pain-free ambulation (Fig. 5). Patients function and satisfaction were evaluated both with the AOFAS scale and by descriptive means, to construct a complete understanding of the pain, function and satisfaction from the patient perspective. The descriptive evaluation, although subjective and unmeasurable, was valuable to overcome cultural and sociodemographic differences that could distort the AOFAS score.

Data were collected and analyzed using SPSS 25.0 (Chicago, IL). Raw data were presented in the form of absolute numbers and percentages, while averages were presented with a standard deviation and a range. A paired-sample t-test was used to compare AOFAS scores. A P-value of less than 0.05 was considered statistically significant.

## Results

All the feet were plantigrade after the procedure and before the cast was fitted. There were no major complications during the postoperative period, and all patients were discharged from the hospital. All 12 patients had a follow-up duration of at least three months, at which point the cast and pins had been removed. Four patients were lost to follow-up. Two patients completed one year of follow-up and did not return for further clinic visits. Eight patients returned for a follow-up visit 3 and 5 years after the procedure. At each patient’s final follow-up visit, all 14 feet were plantigrade with good alignment, and the patients reported improvement in daily activity.

The mean AOFAS score before surgery was 40.58 ± 2.02 (range 40–47). All patients have denied any pain, which added 40 points to their preoperative AOFAS scores and, in most cases, was the only factor that raised the score from a single-digit value. At three years of follow-up, the mean AOFAS score of eight patients with available data had improved to 83.75 ± 4.65 (range 77–90) points (an improvement of 42.88 ± 3.91 points, 95% CI 39.61 to 46.14, *P* < 0.01).

At follow-up, they presented with plantigrade feet. In a few cases, we could notice a slight drop-foot appearing foot structure that was attributed to the scarring associated with the surgical approach. Nevertheless, these feet enabled a more functional gait that was faster and smoother. No recurrence of the deformity was observed at the last follow-up examination of any of our patients. Outcome was not associated with the initial pathology (polio or congenital TEV).

## Discussion

Fixed TEV is a complex deformity that severely impairs mobility. That, in turn, has far-reaching implications for all aspects of daily life, including the ability to commute, study, or work. In developing countries, these limitations can impair the ability to lead an independent and self-sustaining life, putting a strain on the family. While new, sophisticated, and often expensive operative modalities have been demonstrated as effective tools for correcting these deformities, patients living in countries with limited medical access cannot benefit from such “advancements.” This study presents a basic “low-tech” surgical technique for correcting rigid TEV feet and restoring the ability to walk independently. Patients presented to our team with deformities that significantly affected gait and general function. Their deformities forced them to walk on the lateral aspect of their feet, which imposed a slow, disturbed and painful gait. Feet were often non-shoeable. This complex of functional limitation affected many aspects of their lives. Following surgery, although patients did not have a completely normal gait, nor could they qualify for sporting activities, their feet were shoeable and they were able to walk in a way that enabled normal daily activities. The patients also reported that the improvement in their mobility has helped them find work and maintain financial stability.

A few authors have previously described surgical techniques for treating rigid TEV [8, 9, 13]. These are classified based on the type of surgery (bony or soft tissue) and the type of fixation, which can be either internal (plates, screws) or external (K-wires, Steinmann pins, or external fixators). It is common to use a combination of these procedures. The treatment strategy is determined by the configuration of the deformity and its specific components, the patient's needs and preferences, and the surgeons' experience. When developing a treatment strategy, it is critical to consider the local healthcare capabilities in terms of surgical conditions, follow-up availability, and access to rehabilitation. Recent literature indicates that external fixators are becoming the modality of choice for rigid TEV, with promising results [1, 14–17]. Nonetheless, these modalities are expensive and require a high level of surgical expertise as well as strict follow-up and rehabilitation plans. As a result, this technique was inapplicable for treating our patients in developing countries. Another salvage alternative for fixed clubfoot is talectomy [18]. This procedure allows to orient the calcaneopedal unit properly under the tibiofibular mortise. The reduction obtained is maintained by Kirschner wires for 6 weeks before being removed. This procedure is relatively inexpensive and corrects the deformity while enables to avoid possible vascular complications during reduction.

Bony procedures range from single-stage corrective osteotomies to triple arthrodesis. The Cole osteotomy is a lateral closing wedge. The first cut is made vertically, near the center of the navicular and cuboid bones. The second cut is based on the dimensions of the wedge to be removed, beginning anterior to the first cut and connecting the plantar edge [19]. Japas et al. described another technique [20] that employs an anteromedial approach and a V-shape osteotomy between the midtarsal and tarsometatarsal joints, with its apex proximal and at the base of the deformity center (near the navicular). This technique shortens the foot and eliminates the slow healing process of the osteotomy [20]. For the forefoot deformity, Japas et al. described the tarsometatarsal truncated-wedge arthrodesis [20]. The goal of the treatment is to restore the plantigrade foot to allow mobility. In our series of patients, osteotomy was indicated during the preoperative evaluation because the deformities were fixed. Due to the subtalar involvement and the center of the deformity being in the midfoot, a variation of Cole osteotomy [19, 20] was performed. The modification of the technique includes a subtalar approach within the same skin incision and the midtarsal bone. At the final follow-up visit, all patients displayed a plantigrade foot and improved mobility.

Soft tissue procedures aim to prevent deformity progression by reorienting, shortening, or lengthening tendons and ligaments, which can have a balancing effect on distorted joints [21]. Before conducting these procedures, it is essential to assess the involved tendons with a thorough physical examination to determine their competency. Hindfoot equinus originates from the tibiotalar joint and can be addressed by a TAL procedure. The tibialis anterior (TA) tendon functions as a midfoot supinator, dorsiflexor, and forefoot adductor. The transfer of this tendon to the peroneus tertius, or the fifth metatarsal base [21–24], eliminates its effect as a supinator and dorsiflexor while improving the peroneus' ability to pronate and evert the foot into a more plantigrade position. In our series, 10 (83.3%) patients underwent TAL in addition to bony procedures, and none underwent a TA transfer. Although not implemented in this series, a posterior release could be added in cases where both an osteotomy and TAL procedure were not sufficient in correcting the equinus.

In developing countries, such as Vietnam, medical equipment is scarce. The procedures were conducted without fluoroscopy, plates, screws, or external fixators. Furthermore, postoperative physical therapy is frequently unavailable to patients. Traditionally, treatment of fixed clubfoot deformities includes extensive soft tissue release in children and osteotomies in older patients [1, 8–11]. All of the patients in our study had mature bones with fixed deformities. As a result, a combination of soft tissue and bony procedures was carried out, as shown in Table 1. Many studies have described various fixation methods for osteotomies [1, 8–10]. We used Steinmann pins and casts for fixation. Despite using these antiquated methods, all surgeries resulted in fixed plantigrade feet, and all patients reported improved mobility and quality of life. One disadvantage of this technique is the lengthy period of cast immobilization. The non-weight-bearing period in our series was 1.5 months in a circular cast and an additional 1.5 months in a removable walking boot. We could have shortened the duration of immobilization by using new locking plates and/or screws, allowing patients to switch to a boot after only six weeks. Another disadvantage is the inability to perform pre-planned monitored corrections, which are possible when using more modern techniques (Table 2).

**Table 2:** Pre- and post-operative AOFAS scores of patients who underwent surgery for rigid talipes equinovarus (*n* = 8)

No	Surgery	Pain	Function							Alignment	Total
			Maximum walk distance	Gait abnormality	Hindfoot motion (inversion plus eversion	Ankle-hindfoot stability (anteroposterior, varus-valgus)	Function-activity limitations/support requirements	Walking surfaces	Sagittal motion (flexion plus extension)		
1	Pre	40	0	0	0	0	0	0	0	0	40
	Post	40	5	8	0	8	7	5	0	10	83
2	Pre	40	0	0	0	0	0	0	0	0	40
	Post	40	5	4	0	8	7	3	0	10	77
3	Pre	40	0	0	3	0	0	0	4	0	47
	Post	40	5	8	3	8	7	5	4	10	90
4	Pre	40	0	0	0	0	0	0	0	0	40
	Post	5	8	3	8	7	3	0	10	84	
5	Pre	40	0	0	0	0	0	0	0	0	40
	Post	40	5	5	5	8	7	3	4	10	87
6	Pre	40	0	0	0	0	0	0	0	0	40
	Post	40	5	4	3	8	7	5	4	10	86
7	Pre	40	0	0	0	0	0	0	0	0	40
	Post	–	–	–	–	–	–	–	–	–	–
8	Pre	40	0	0	0	0	0	0	0	0	40
	Post	40	5	4	3	8	7	5	4	10	86
9	Pre	40	0	0	0	0	0	0	0	0	40
	Post	40	5	4	0	8	7	3	0	10	77
10	Pre	40	0	0	0	0	0	0	0	0	40
	Post	–	–	–	–	–	–	–	–	–	–
11	Pre	40	0	0	0	0	0	0	0	0	40
	Post	–	–	–	–	–	–	–	–	–	–
12	Pre	40	0	0	0	0	0	0	0	0	40
	Post	–	–	–	–	–	–	–	–	–	–

There are several limitations to this study. The short duration of follow-up for some of the patients is probably the most problematic aspect of this study. Another significant issue was the lack of serial physical therapy, which would have been a significant aspect of treatment in any other setting. We did not evaluate the different types of deformities or the degree of improvement. Overall improvement was measured in terms of mobility and plantigrade foot maintenance. There was no radiographic follow-up available. Although it has been argued in the past that there is only a weak correlation between radiographic findings and clinical outcomes [25], if this modality had been readily available, we would have used it to further evaluate the surgical outcomes. Another significant issue was that none of the patients reported any pain before or after the surgery, which could be attributed to a Vietnamese cultural trait. This proclivity affected the AOFAS results, increasing the score by at least 40 points in all tests. Furthermore, scores obtained during follow-up visits or by phone may have been higher due to the patients' willingness to express gratitude to their caregivers. The AOFAS score had been developed in western countries where demographic, cultural, and socioeconomic features are inherently different than in developing countries. This difference can influence health perception, and health services-related expectations, and deeply affect patient-reported outcome scores. Moreover, the inaccessibility to medical care, and the difference that exists between a surgeon–patient relationship in a regular hospital and in a humanitarian mission could significantly distort validated scores. Despite this limitation, it is important to have an evaluation of the functional outcomes, and the extent at which these surgeries improved patient’s quality of life. A more descriptive approach for evaluating patient’s function before and after surgery could add significant and valuable information and could allow a better understanding of the value of these procedures in underserved and remote communities. We therefore added to the results a description of the way patients have been affected by their pathology both before and after surgery. This approach aimed to allow a more profound description of patients function and satisfaction, with both measurable and non-measurable means (Table 3).

**Table 3:** Postoperative AOFAS scores of patients who underwent surgery for rigid talipes equinovarus (n = 12)

No	Pain	MWD	GA	HM	AHS	FAL	WS	SM	Al	Total
1	40	5	8	0	8	7	5	0	10	83
2	40	5	4	0	8	7	3	0	10	77
3	40	5	8	3	8	7	5	4	10	90
4	40	5	8	3	8	7	3	0	10	84
5	–	–	–	–	–	–	–	–	–	–
6	40	5	4	3	8	7	5	4	10	86
7	40	5	5	5	8	7	3	4	10	87
8	40	5	4	3	8	7	5	4	10	86
9	40	5	4	0	8	7	3	0	10	77
10	–	–	–	–	–	–	–	–	–	–
11	–	–	–	–	–	–	–	–	–	–
12	–	–	–	–	–	–	–	–	–	–

## Conclusion

Using low-technical surgical modalities, we were able to achieve plantigrade and walkable feet in patients with rigid equinovarus.

## Data Availability

The datasets used and/or analyzed during the current study are available from the corresponding author on reasonable request.
